# DNA vaccination with a gene encoding *Toxoplasma gondii* Deoxyribose Phosphate Aldolase (TgDPA) induces partial protective immunity against lethal challenge in mice

**DOI:** 10.1186/1756-3305-7-431

**Published:** 2014-09-08

**Authors:** Ibrahim A Hassan, Shuai Wang, LiXin Xu, RuoFeng Yan, XiaoKai Song, Xiangrui Li

**Affiliations:** College of Veterinary Medicine, Nanjing Agricultural University, Nanjing, Jiangsu 210095 People’s Republic of China

**Keywords:** Toxoplasma gondii, Deoxyribose phosphate aldolase, DNA vaccines

## Abstract

**Background:**

*Toxoplasma gondii* is an obligate intracellular parasite that causes a pathological status known as toxoplasmosis, which has a huge impact on human and animal health. Currently, the main control strategy depends on the usage of drugs that target the acute stage of the infection, however, drawbacks were encountered while applying this method; therefore, development of an alternative effective method would be important progress. Deoxyribose Phosphate Aldolase (TgDPA) plays an important role supporting cell invasion and providing energy for the parasite.

**Methods:**

TgDPA was expressed in *Escherichia coli* and the purified recombinant protein was used to immunize rats. The antibodies obtained were used to verify *in vitro* expression of TgDPA. The vector pVAX1 was utilized to formulate a DNA vaccine designated as pTgDPA, which was used to evaluate the immunological changes and the level of protection against challenge with the virulent RH strain of *T. gondii*.

**Results:**

DNA vaccine, TgDPA revealed that it can induce a strong humoral as well as cellular mediated response in mice. These responses were a contribution of T_H_1, T_H_2 and T_H_17 type of responses. Following challenge, mice immunized with TgDPA showed longer survival rates than did those in control groups.

**Conclusions:**

Further investigation regarding TgDPA is required to shed more light on its immunogenicity and its possible selection as a vaccine candidate.

## Background

*Toxoplasma gondii* is an important zoonotic parasite infecting a wide range of warm blooded hosts, causing a pathological condition known as toxoplasmosis [1]. The infection is mainly acquired either by using water contaminated with oocysts released by the final host or handling intermediate host tissues infested with the asexual cysts [2, 3]. In humans, there are two types of the infection according to symptoms; the first type is the asymptomatic form, resulting in a latent infection with tissue cysts. This form is less frequently seen in immunologically intact individuals. However, the infection could be severe in specific groups of patients, such as immunologically impaired individuals (AIDS or organ transplants) or congenitally infected fetuses and newborns [4, 5].

Currently, the strategies of toxoplasmosis control mainly rely on the application of chemotherapeutics targeting the acute phase of the infection, however, some drawbacks were found to be associated with drug application, e.g; rapid re-infection besides toxic effects of the drugs [6, 7]. Such issues ‘blew the whistle’ , shifting the research directions into the area of vaccine development as an alternative control strategy for toxoplasmosis, with DNA vaccines receiving considerable attention [6].

Recent important progress has been made identifying anti-toxoplasma vaccine candidates that can stimulate an immunological response, with most of the work focusing on tachyzoite surface antigens, namely SAG1, SAG2 and SAG3, and SAG1 was recognized to be the most promising candidate in this group [8–11]. In the same context, *T. gondii* excretory secretory antigens like GRA molecules, have also been reported to demonstrate significant immunogenic capabilities [12–14]. Vaccination with DNA vaccines has been found to induce effective humoral and cellular immune responses, with both CD4^+^ T helper cells and CD8^+^ cytotoxic T cells included in these responses [15]. Such elements are important for understanding the mechanisms through which the parasite modulates the host immune response during both acute and chronic phases of the disease [16].

Deoxyribose phosphate aldolase, a glycolytic enzyme, functionally mediates in host cell invasion, acting as a bridge linking actin filaments to the parasite’s surface adhesion microneme protein 2. Furthermore, aldolase plays an essential role providing carbon and energy sources for the organism, as part of the glycolysis cycle, on which the parasite gliding motility depends during the invasion process [17–20].

Blocking the parasite from invading the cell and consequently preventing the parasite form multiplying may help in reducing the parasitic burden and leave the parasite exposed to other immunological elements, thus in this study we demonstrated the immunological changes after vaccination of mice with a DNA vaccine encoding TgDPA followed by challenge with virulent *T. gondii* RH strain.

## Methods

### Animals and parasite

Six to eight week-old female Swiss Webster (SW) mice were purchased from The Center of Comparative Medicine, Yangzhou University (Yangzhou, China) and maintained under specific-pathogen-free standard conditions. All animal experiments were approved by the Animal Ethics Committee of Nanjing Agricultural University (Approval number 200709005). *Toxoplasma gondii* strain RH (Type I), was provided by The Laboratory of Veterinary Molecular and Immunological Parasitology, Nanjing Agricultural University, China. To maintain the parasite, as described by [21], intraperitoneally injected SW mice were infected with the parasite tachyzoites. Every 3 days, the tachyzoites were harvested and recovered from peritoneal washings of infected mice to be used for re-infection.

### Construction of the prokaryotic plasmid

According to the manufacturer's protocol Trizol reagent (Takara, Life Technologies), total RNA of *T. gondii* was extracted from *T. gondii* tachyzoites, followed by construction of the cDNA. The open reading frame (ORF) of **D**eoxyribose **P**hosphate **A**ldolase (TgDPA) gene (XM_002365690.1) was obtained from *T. gondii* cDNA by PCR amplification using the following synthetic primers in which recognition sites were inserted as underlined below.

DPA: Forward primer: 5’- T*GGATCC*ATGGATGCAGAACAACAGG-3’ (*BamH* I).

Reverse primer: 5’- GC*AAGCTT*TTACAGAACGAATTCCCGG-3’ (*Hind* III).

The PCR product of TgDPA was inserted into the pMD-18 T Vector (TaKaRa) to generate prokaryotic plasmid pMD-TgDPA. The recombinant plasmid was used to transform the bacteria *E. coli* DH5α (JM109). Insertion was confirmed by sequencing in both directions. After purification, pMD-TgDPA recombinant plasmid was double digested with appropriate restriction enzymes (*BamH* I/*Hind* III) and sub-cloned into the matching sites of pET28a (+) vector (Novagen). Following the screening by enzymatic cleavage, the positive clones were sequenced in both directions to ensure the plasmid designated as pET28a/TgDPA was successfully constructed.

### Expression and purification of TgDPA recombinant protein

The recombinant plasmid designated as pET28a/TgDPA was used to transform *E. coli* bacteria strain BL21 (DE3), and protein expression was induced by addition of 0.8 mM Isopropyl-β-D-thiogalactopyranoside (IPTG) after the OD_600_ of the bacterial culture reached 0.6 at 37°C. The cells were incubated at 37°C for 5 hr and harvested by centrifugation. The cell pellet was lysed using lysozyme (10 μg/ml) followed by disruption of the cells using sonication. Expression of the protein was analyzed by 12% (w/v) Sodium Dodecyl Sulfate Polyacrylamide Gel Electrophoresis (SDS-PAGE).

The recombinant protein was purified by Ni^2+^-nitrilotriacetic acid (Ni^2+^-NTA) column (GE Healthcare) according to the manufacturer’s instructions. Purity of the protein was detected by 12%SDS-PAGE. The concentration of the protein was determined according to the Bradford procedure using bovine serum albumin (BSA) as a standard. The purified protein was used to develop anti-rTgDPA sera and the rest of the protein was stored at −20°C for later applications.

### Construction of the eukaryotic plasmid

The restriction enzymes *BamH* I and *Xho* I were used to digest the recombinant plasmid pET28a/TgDPA and the target gene DNA fragment was directionally sub-cloned into the pVAX1 vector (Invitrogen, Life Technologies), which was previously linearized with similar enzymes. The resultant recombinant plasmid designated pTgDPA was verified and confirmed by sequencing in both directions and also with double enzyme digestion. Plasmids were then purified from transformed *E. coli* DH5 (JM109) cells by anion exchange chromatography (EndoFree Plasmid mega Kit Qiagen) following the manufacturer’s instructions, dissolved in sterile endotoxin-free H_2_O and the concentration was determined by spectrophotometer at OD_260_ and OD_280_. The recombinant plasmid was stored at −20°C until use.

### Plasmid *in vitro*translation of pTgDPA

BHK cells were cultured in Dulbecco’s modified Eagle’s medium (DMEM, GIBCO) with 10% Fetal Bovine Serum (FBS), 100 mg/ml streptomycin and 100 IU/ml penicillin at 37°C in the presence of 5% CO_2_. Before transfection, BHK cells were transferred in a 6-well plate (Costar, USA). When the confluency of the cells reached 80%-90%, 5 μg of the recombinant eukaryotic plasmid (pTgDPA) was used to transfect the cells using Lipofectamine 2000 regent (Invitrogen) according to the manufacturer’s guidance. The empty vector pVAX1 (5 μg) was also transfected into BHK cells as a negative control. Lipofectamine 2000 reagent was respectively mixed with pTgDPA and pVAX1 at a concentration of 10 μg/ml in DMEM without Fetal Bovine Serum (FBS) and antibiotics, and was incubated at room temperature for 30 min. The mixture of lipofectamine and plasmid was then added into BHK cells. The cells were incubated with the transfection mix for 6 hr at 37°C in the presence of 5% CO_2_. At the end of incubation, fresh growing medium was supplemented and plates were returned to the cell incubator for further incubation. After 48 hr of incubation, BHK cells were collected and expression of the gene was evaluated by Western blotting analysis.

### Mice immunization and challenge

To observe the immunogenicity of DPA, mice were randomly divided into four groups (25/group). Before vaccination, plasmids were diluted and suspended in sterile phosphate buffered saline (PBS pH 7.4) to a final concentration of 100 μg/ml. All experimental groups were injected intramuscularly (quadriceps muscle), tw{Capron, 1988 #496}o times at weeks 0 and 2. Control groups received PBS or empty plasmid or no treatment. Blood samples of mice were collected before vaccination (negative control), as well as at the time of the second injection and 2 weeks after the second injection. The sera were stored at −20°C for antibody evaluation and cytokine measurement. Two weeks after the last injection, the mice in the four groups were challenged intraperitoneally (i.p) with 2 × 10^4^ tachyzoites of *T. gondii* RH strain. The survival times of the mice were observed and recorded on a daily basis.

### Determination of antibodies and isotype distribution by ELISA

Enzyme-linked immunosorbent assay, ELISA technique, was used to determine the levels of total IgG in sera samples collected at week 0, 2 and 4, while levels of IgA, IgM, IgE and subclasses IgG_1_ and IgG_2a_, were determined in sera samples collected at week 4. The microtiter plates (Costar, USA) were coated with 5 μg rTgDPA recombinant protein in 50 mM carbonate buffer (pH 9.6) and incubated at 4°C overnight. After three washes, the plates were blocked with 2% skimmed milk for 1 h at 37°C and subsequently incubated with the mouse sera diluted (1:100) in the same blocking buffer for 1 h at 37°C. HRP-conjugated goat anti-mouse of IgA, IgM, IgE, IgG, IgG_1_ and IgG_2a_ (Santa Cruz Biotechnology) were used as secondary antibody (1:1000). Finally, the immune complexes were developed by incubating with 3,3,5,5-Tetramethylbenzidine (TMB) for 30 min. The reaction was stopped by adding 2MH_2_SO_4_, and the absorbance was measured at 450_nm_ with an automated ELISA reader (MULTISKAN FC, Thermo scientific), all samples were run in duplicate.

### Assay of cytokines

To assay cytokine production levels, sera from each experimental group were obtained as described previously. Interferon Gamma (IFN-γ), Interleukin-4 (IL-4), Interleukin-17 (IL-17) and Transformation Growth Factor-β1 (TGF-β1) were measured using ready ELISA kits according to the manufacturer’s instructions (Boster Systems, Wuhan, China). Cytokine concentrations were determined by reference to standard curves constructed with known amounts of mouse recombinant IL-4, IL-17, IFN-γ and TGF-β1. The analysis was performed with the data from three independent experiments.

### Flow cytometry analysis of T cell subsets and MHC molecules

The percentages of T cells subsets CD4^+^ and CD8^+^, beside MHC-I and MHC-II molecules in the spleenocytes of mice in the test groups, pTgDPA and pVAX1, PBS and blank, were analyzed using the flow cytometry technique as described by [22].

Splenocytes suspensions (1 × 10^6^ cells/ml) were dually stained with anti-mouse CD3e-FITC + anti-mouse CD8-PE, anti-mouse CD3e-FITC + anti-mouse CD4-PE, anti-mouse CD3e-FITC + anti-mouse MHC-I-PE or anti-mouse CD3e-FITC + anti- mouse MHC-II-PE (eBioscience) for 30 min at room temperature in the dark. Cell population analysis was conducted by FACScan flow cytometry with CellQuest software (BD Biosciences, Franklin Lakes, NJ, USA). A lymphocyte specific gating was set according to forward and side scatters profiles. The percentages of CD4^+^ and CD8^+^ T lymphocytes, MHC-I and MHC-II molecules in mice spleenocytes were determined as described by [23].

### Statistical analysis

All statistical analyses were performed by Graphpad Prism 5.Ink software. The differences of the data between all the groups were compared by one-way ANOVA. Survival rate of the mice was compared using the Kaplan-Meier method. The results in comparisons between groups were considered different if P < 0.05.

## Results

### TgDPA recombinant plasmids expression

**Figure 1:**
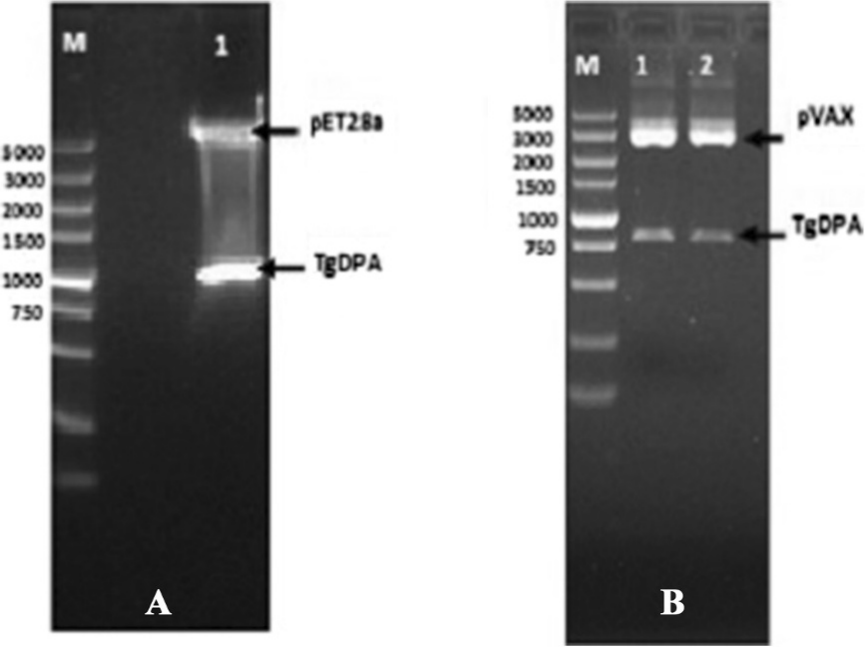
**Recombinant plasmids of TgDPA. (A)** Lane (1) the prokaryotic construct pET28/TgDPA was double digested by *Bam* I and *Hind* III enzymes and the product was resolved by 1% agarose gel to verify a band of size 807 bp. (M) Represents DNA Molecular marker. **(B)** Lanes 1 & 2, the eukaryotic construct pVAX1/TgDPA was double digested by *BamH* I and *Xho* I enzymes and the product was resolved by 1% agarose gel to verify a band of size 807 bp. (M) Represents DNA Molecular marker.

**Figure 2:**
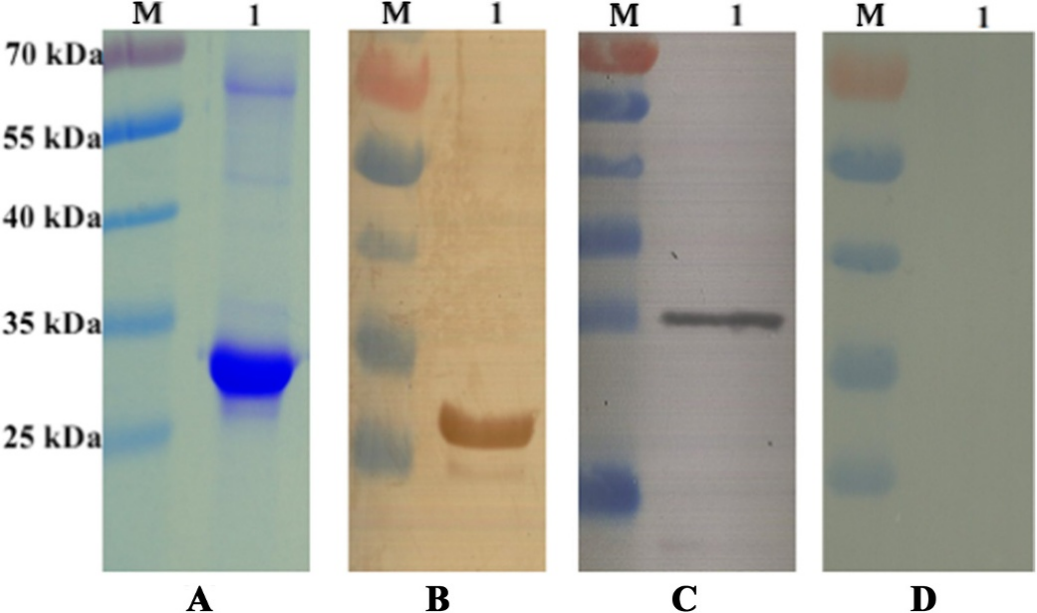
**Identification of TgDPA expression in** ***E. coli*** **BL21 (DE3) by SDS-PAGE and western-blotting. (A)** Purified recombinant TgDPA protein was resolved by 12% SDS-PAGE gel and stained with coomassie brilliant blue R250. **(B)** Western blot of rTgDPA recombinant product probed with sera of rats experimentally immunized with rTgDPA. **(C)** Western blot of pVAX1/TgDPA expressed in BHK cells probed with anti-rTgDPA antibodies. **(D)** No band equivalent to TgDPA was observed in the negative control BHK cells transfected with the empty pVAX1 vector. (M) Represents Pre-stained protein marker.

Following sequence analysis confirming that the TgDPA DNA fragment was directionally inserted; pET28a/TgDPA was successfully constructed (Figure 1A). Expression of the recombinant protein took place by IPTG induction, followed by purification, the product was analyzed using SDS-PAGE (Figure 2A). The purified protein was used to develop polyclonal antibodies (anti-rTgDPA) and western blotting technique was used to verify the results (Figure 2B).

As for the eukaryotic plasmid pTgDPA, a separate trial was conducted to screen *in vitro* expression. Lysates of the BHK cells transfected with pTgDPA were probed with anti-rTgDPA polyclonal antibodies revealing successful expression of the protein (Figure 2C), while cells transfected with empty pVAX1 exposed no specific bands and remained negative (Figure 2D).

### Antibody response in immunized mice and subclass determination

**Figure 3:**
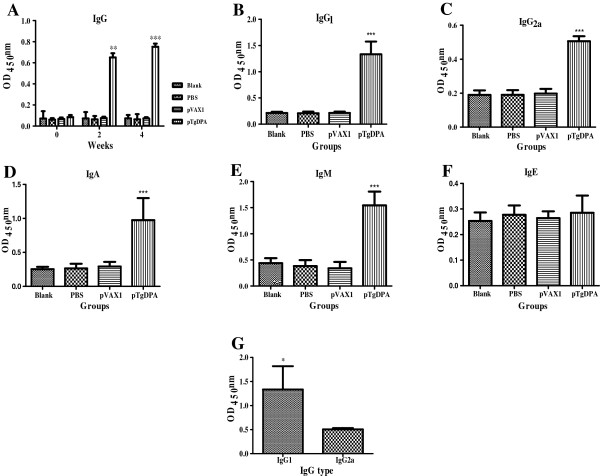
**Specific antibody response induced by DNA immunization with pTgDPA compared to pVAX I, PBS and blank controls using indirect ELISA. (A)** Total IgG was evaluated in sera samples collected at 3 time points marked as week 0, week 2 and week 4 (n = 5). levels of: **(B)** IgG_1_ **(C)** IgG_2a_, **(D)** IgA, **(E)** IgM and **(F)** IgE in sera samples collected at week 4 of the experiment (n = 5). **(G)** Comparison of the distribution levels of IgG_1_ and IgG_2a_ subclasses in sera of pTgDPA vaccinated group after the booster dose. In all experiments, comparison results were expressed as means ± SD of OD_450_. The asterisk designates statistically significant differences (p < 0.05) between groups.

The titers of total IgG, beside subclasses IgG_1_ and IgG_2a_ were measured prior to and after immunization, using standard ELISA. As shown in Figure 3A, specific total IgG antibodies were detected in the experimental group vaccinated with pTgDPA. There was a significant difference at (p < 0.05) between pTgDPA group after first immunization (0.651 ± 0.04) and 2^nd^ immunization (0.752 ± 0.03), compared to the control groups of pVAX1 (0.073 ± 0.011), PBS (0.050 ± 0.07) and Blank (0.07 ± 0.03).

IgG isotype determination revealed that, both IgG_1_ (1.33 ± 0.485) and IgG_2a_ (0.506 ± 0.029) were significantly (P < 0.05) stimulated after delivering the antigen (Figure 3B and C). Moreover, the difference between the levels of these isotypes was found to be significant at (P < 0.05), for the advantage of IgG_1_ (Figure 3G). Regarding IgA, IgM and IgE, and when compared to the control groups, dynamics of the first two antibody types demonstrated high OD values (P < 0.05) in the immunized group (0.974 ± 0.33) and (1.55 ± 0.26) respectively (Figure 3D and E). However, IgE activity showed no significant changes at the time of evaluation (Figure 3 F).

### Cytokine production

**Figure 4:**
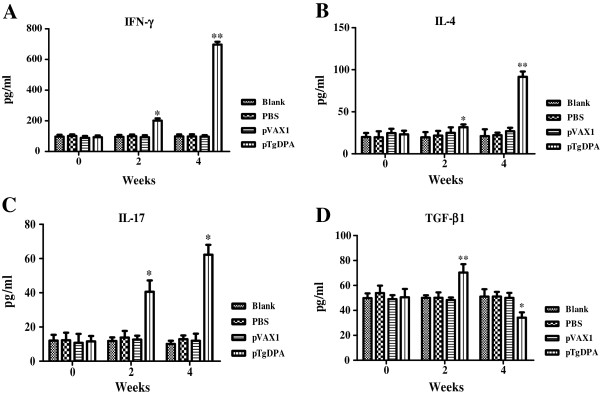
**Cytokine production.** Antibody-captured ELISA was used to determine the production levels of **(A)** IFN-γ, **(B**) IL-4, **(C**) IL-17 and **(D**) TGF-β1, in sera samples (n = 5) collected at weeks 0, 2 and 4, and the comparison results were expressed as means ± SD of pg/ml. The asterisk designates statistically significant differences (p < 0.05) between groups. Results presented here were from three independent experiments.

Sera samples collected at weeks 0, 2 and 4 were used to measure the amounts of IFN-γ, IL-4, IL-17 and TGF-β1 produced in the different experimental groups. As shown in Figure 4A, mice vaccinated with pTgDPA generated significant levels of IFN-γ at (P < 0.05) compared to mice in the control groups, peak production was reached 2 weeks after the last immunization (697.0 ± 8.39).

IL-4 and IL-17 of the pTgDPA group showed a significant difference (P < 0.05) against the control groups (Figure 4B and C). The peak of production was also at 2 weeks after the booster dose (91.6 ± 1.34) for IL-4 and (62.2 ± 2.83) for IL-17. Additionally, TGF-β1 (Figure 4D) displayed a different activity. Immunized groups showed a significant peak after the first immunization (70.4 ± 6.66), which was dramatically decreased (34.2 ± 2.26) two weeks after the last immunization. Compared to the control groups both time points were significant at (P < 0.05).

### Recruitment of T lymphocytes subpopulations and MHC molecules

**Figure 5:**
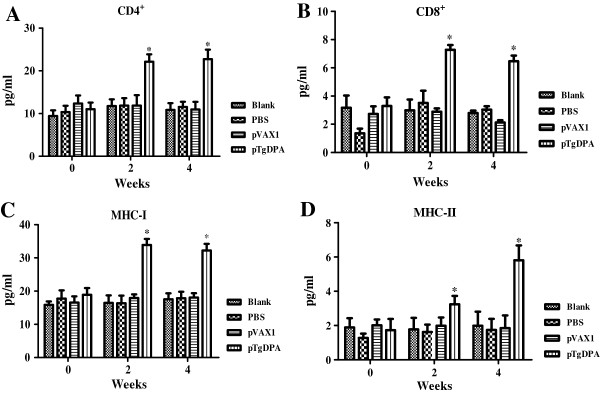
**Quantification of T lymphocytes and MHC molecules using flow cytometry analysis.** Harvested at weeks 0, 2 and 4 (n = 5), splenocytes were used to quantify; **(A)** CD4^+^ T cells. **(B):** CD8^+^ T cells. **(C):** MHC-I molecules. **(D)** MHC-II molecules. Data were represented as means ± SD. The asterisk designates the significant differences (p < 0.05) between the groups. Results presented here were from three independent experiments.

**Figure 6:**
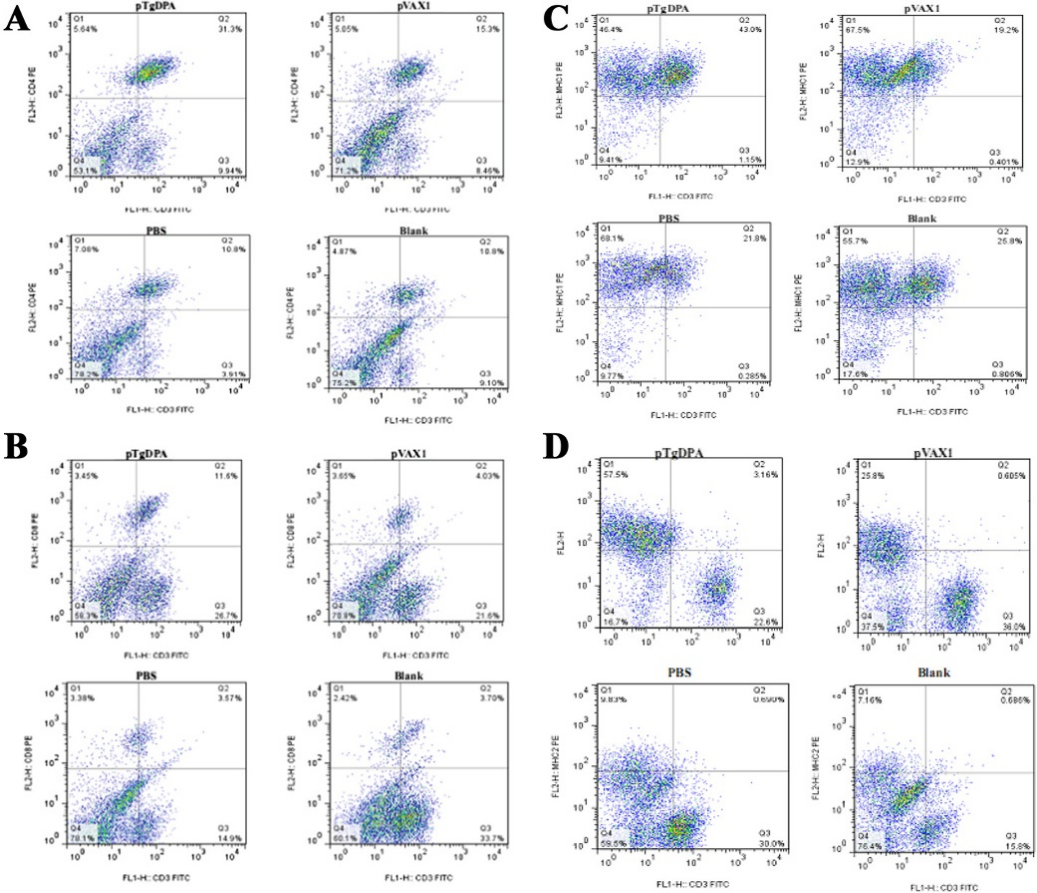
**Flow cytometry strategy.** Detection of T lymphocyte subpopulation and MHC molecules using flow cytometry technique (CD3 gated), **(A)** CD4^+^ T lymphocytes (CD3^+^CD4^+^, region Q2). **(B)** CD8^+^ T lymphocytes (CD3^+^CD8^+^, region Q2). **(C)** MHC-I molecules (CD3^+^MHC-I, region Q2). **(D)** MHC-II molecules (CD3^+^MHC-II, region Q2).

As shown in (Figures 5A & 6A), following immunization with pTgDPA, the percentage of CD4^+^ T cells was significantly increased (P < 0.05) in the pTgDPA immunized group at week 4 (22.74 ± 2.23), compared with that in pVAX1 group (12.34 ± 1.90), PBS group (10.36 ± 1.46) and the blank group (9.44 ± 1.33).

As for CD8^+^ T cells, significant differences (P < 0.05) were also detected among the different experimental groups at 2 weeks after the last immunization. TgDPA group showed the highest percentage (6.47 ± 0.40), while pVAX1, PBS and blank control group remained at low levels as (2.13 ± 0.16), (3.04 ± 0.24) and (2.80 ± 0.17) respectively (Figures 5B and 6B).

After both prime and booster immunizations, MHC-I molecules of the immunized group displayed sustained high significant readings (33.89 ± 1.83) and (32.22 ± 1.98) in contrast to pVAX1 (18.04 ± 1.34), PBS (17.85 ± 1.98) and Blank (17.56 ± 1.78) groups (Figures 5C & 6C). Concerning MHC-II molecules, a gradually increasing pattern was noticed in the vaccinated group (Figures 5D & 6D) starting at week 2 of the experiment reaching a peak point (5.81 ± 0.87) at week 4. Compared to control groups (1.85 ± 0.74), (1.74 ± 0.65) and (1.99 ± 0.82) the difference between these values was found significant at (P < 0.05).

### Protection of vaccinated mice against challenge with *T. gondii*RH strain

**Figure 7:**
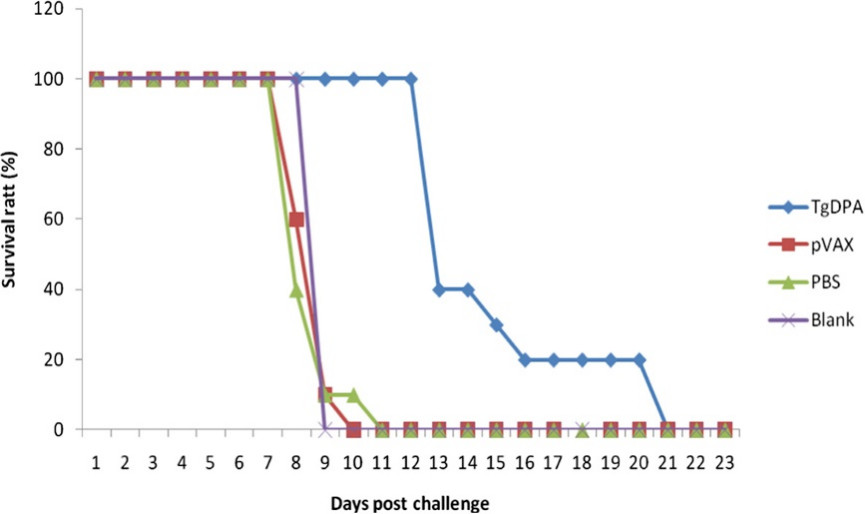
**Survival curves of mice in pTgDPA, pVAX1, PBS and Blank groups, following challenge with** ***T. gondii*** **RH strain.** Survival time was monitored daily after lethal challenge with 2 × 10^4^ tachyzoites of virulent *T. gondii* RH strain, 2 weeks after the last immunization. The mice immunized with pTgDPA were dead from day 11 to day 20, showing an increased survival time compared with mice in the control groups (pVAX1, PBS, blanking controls), which died within 8–9 days after challenge (P < 0.05).

In order to evaluate the protective effect of pTgDPA DNA vaccine against acute toxoplasmosis, vaccinated and control mice groups were challenged with lethal *T. gondii* tachyzoites within the second week after booster immunization. Mortality was observed daily until all the mice died and survival curves of different groups were generated and are shown in (Figure 7). Significantly longer survival time (20 days) was observed in mice immunized with pTgDPA against the control group, who died within 8–9 days after challenge (p < 0.05).

## Discussion

In this study, we have demonstrated that a DNA vaccine encoding DPA of *T. gondii* could elicit a considerable specific immune response, as well as providing significant levels of protection against *T. gondii* challenge.

In this report, humoral response was analyzed after vaccination with TgDPA. Immunized mice generated specific high titers of IgG, in contrast to control groups. Analysis of IgG isotypes revealed that the levels of IgG_1_ were significantly higher than that of IgG_2a_. Similar results were detected after *T. gondii* antigens like cathepsin proteases, protein kinase 3 and GRA4 were evaluated [24–26]. IgG_2a_ and IgG_1_ are characteristic of T_H_1 and T_H_2 immunity, respectively [27]. Higher IgG_1_ levels in this research indicated that TgDPA induced mainly T_H_2 responses.

Immunoglobulins IgA, IgM and IgE were reported to participate in the immunological responses against *T. gondii* infection. However, less attention has been placed on these immunoglobulins during vaccination trials against *T. gondii* [28, 29]. IgA is an important immunoglobulin to act on neutralization of toxins and pathogenic microbes, beside regulating interaction between specific receptors and immune mediators [30, 31]. With specific relation to *T. gondii* infection, IgM was reported to enhance the phagocytic capacity of neutrophils and activate the complement cascade which might result in killing of *T. gondii* as well as reducing the spread of *T. gondii* by blocking cell invasion [32–34]. In our research, high titers of IgA and IgM were detected in the immunized group. This suggested that both IgA and IgM played roles in the protective responses induced by the TgDPA.

IgE was recognized during the infection of toxoplasmosis [35, 36]. However, our data revealed no significant traces of this immunoglobulin after vaccination with TgDPA. Our results were consistent with previous studies [37].

IFN-γ is a key cytokine of T_H_1 type immune response and is known to play an important role in resistance against *T. gondii*. This cytokine supports many immunological mechanisms, interferes with survival and multiplication of intracellular pathogenic organisms and leads to the eradication of pathogenic organisms [38–42]. Remarkable levels of IFN-γ were detected in this study. This result and the release of IgG_2a_ isotype substantiated the involvement of T_H_1 response against TgDPA. This finding agrees with many studies in which significant production of IFN-γ was detected after immunization with *T. gondii* antigens [25, 26, 43–47].

IL-4 is known as an immune regulatory cytokine of T_H_2 type of immune response. In this research, the immunized group showed a significant release of IL-4 compared to the control groups. The positive increase of this cytokine during this investigation agrees with previous reports highlighting the role of this cytokine during vaccination trials using *T. gondii* antigens [48, 49]. The significant release of IL-4, together with the release of IgG_1_ showed that the T_H_2 type response was involved in the protection provided by TgDPA against the *T. gondii* challenge [27, 50, 51].

The T_H_1/T_H_2 immune response pattern has dominated the studies of cell-mediated immune resistance to infections like toxoplasmosis [52]. Recently, a new lineage of T helper cells recognized for producing proinflammatory cytokines, such as IL-17, IL-21, and IL-22 [53], had been identified and designated as T_H_17. Cytokines related to T_H_17 are associated with recruitment and activation of neutrophils during inflammatory diseases [54, 55]. However, evaluation of T_H_17 responses during immunization trials against *T. gondii* received less attention compared to T_H_1 and T_H_2. In this investigation, after the booster immunization, a significant increase of IL-17 concentration was detected. This finding indicated that TgDPA was capable of inducing T_H_17 differentiation and resulted in an inflammatory reaction. This result also showed that T_H_17 response obviously played an important role during immunization with TgDPA.

TGF-β is a typical cytokine of T_reg_ cells and usually plays inhibitory roles in the immune responses [56–59]. The inhibitory function of T_reg_ cells was also demonstrated during toxoplasmosis infection [60]. In this study, the immune group displayed significantly low concentrations of TGF-β1 after the booster immunization compared to the control groups. It indicated that immunization with TgDPA down-regulated T_reg_ cells response. This character of TgDPA will be beneficial to its potential as a vaccine candidate.

Resistance against *T. gondii* parasite is characterized by the induction of specific CD4^+^ and CD8^+^ T cells, which eventually lead to the killing of the parasite [61, 62]. In murine models, CD4^+^ T cells were crucial regulators of the immune response during resistance against toxoplasmosis, while in humans CD4^+^ displayed cytotoxic activity against *T. gondii* infected cells [15, 63, 64]. On the other hand, CD8^+^ subtype were considered to be the major effector cytotoxic T lymphocyte (CTL) cells mediating lysis of *T. gondii* infected host cells [65–67]. In this investigation, our data demonstrated that both cell subtypes were significantly accumulated in response to immunization with TgDPA. This result corresponds with reports regarding immunological responses to *T. gondii* antigens [16, 68–72].

In this study, results that further support the increments of both CD4^+^ and CD8^+^ T cell subsets were the simultaneous significant increases of the ratios of MHC-I and MHC-II. Activation of CD4^+^ T cells requires the endocytosis or phagocytosis of secreted or exogenous proteins entering into the MHC class II pathway, while the activation of CD8^+^ depends on the recognition of antigens restricted by MHC class I molecules [27, 73]. The findings in this study demonstrated that TgDPA antigen was presented through MHC-I and MHC-II.

As a result of these significant immunological changes, pTgDPA vaccinated mice survived for a longer time compared to the control groups in this research. However, due to uncontrolled parasite replication, pTgDPA mice ultimately succumb during late acute infection. It indicated that the DNA vaccine of pTgDPA did not provide complete protection. However, investigations concerning this protein should further be conducted.

One of the prominent advantages of DNA vaccine application is their induction of CTL cells. CTL cells kill the pathogen infected cells mainly by inducing apoptosis [74]. *T. gondii* maintains its survival and replication by interfering with infected cells apoptosis, blocking an important pathway known as caspase cascade [75–78]. Measurement of damaged infected cells was an important tool to measure CTL function [74, 79, 80]. Application of such methods is required in further investigations regarding TgDPA antigen to highlight its role in CTL response stimulation and resistance development against *T. gondii* infection.

## Conclusion

Our study demonstrated that the pTgDPA delivered as a single protein is an antigen with the potential of inducing and regulating significant levels of humoral as well as cellular (T_H_1, T_H_2 and T_H_17) immune responses against acute *T. gondii* infection. This finding may encourage more investigations in evaluating the immunogenicity of DPA based vaccines against Toxoplasmosis.

## References

[1] Montoya JG, Liesenfeld O (2004). Toxoplasmosis. Lancet.

[2] Robert-Gangneux F, Darde ML (2012). Epidemiology of and diagnostic strategies for toxoplasmosis. Clin Microbiol Rev.

[3] Wang L, Cheng H-W, Huang K-Q, Xu Y-H, Li Y-N, Du J, Yu L, Luo Q-L, Wei W, Jiang L (2013). Toxoplasma gondii prevalence in food animals and rodents in different regions of China: isolation, genotyping and mouse pathogenicity. Parasit Vectors.

[4] Weiss LM, Dubey JP (2009). Toxoplasmosis: A history of clinical observations. Int J Parasitol.

[5] Glor SB, Edelhofer R, Grimm F, Deplazes P, Basso W (2013). Evaluation of a commercial ELISA kit for detection of antibodies against Toxoplasma gondii in serum, plasma and meat juice from experimentally and naturally infected sheep. Parasit Vectors.

[6] Rodriguez JB, Szajnman SH (2012). New antibacterials for the treatment of toxoplasmosis; a patent review. Expert Opin Ther Pat.

[7] Bhopale GM (2003). Development of a vaccine for toxoplasmosis: current status. Microbes Infect.

[8] Couvreur G, Sadak A, Fortier B, Dubremetz J (1988). Surface antigens of Toxoplasma gondii. Parasitology.

[9] Lekutis C, Ferguson DJ, Grigg ME, Camps M, Boothroyd JC (2001). Surface antigens of Toxoplasma gondii: variations on a theme. Int J Parasitol.

[10] Chuang S-C, Ko J-C, Chen C-P, Du J-T, Yang C-D (2013). Induction of long-lasting protective immunity against Toxoplasma gondii in BALB/c mice by recombinant surface antigen 1 protein encapsulated in poly (lactide-co-glycolide) microparticles. Parasit Vectors.

[11] Cong H, Zhang M, Xin Q, Wang Z, Li Y, Zhao Q, Zhou H, He S (2013). Compound DNA vaccine encoding SAG1/SAG3 with A2/B subunit of cholera toxin as a genetic adjuvant protects BALB/c mice against Toxoplasma gondii. Parasit Vectors.

[12] Capron A, Dessaint JP (1988). Vaccination against parasitic diseases: some alternative concepts for the detection of protective antigens. Ann Inst Pasteur Immunol.

[13] Cesbron-Delauw M-F (1994). Dense-granule organelles of Toxoplasma gondii: Their role in the host-parasite relationship. Parasitol Today.

[14] Sun X-M, Zou J, Elashram Saeed A, Yan W-C, Liu X-Y, Suo X, Wang H, Chen Q-J (2011). DNA vaccination with a gene encoding Toxoplasma gondii GRA6 induces partial protection against toxoplasmosis in BALB/c mice. Parasit Vectors.

[15] Montoya JG, Lowe KE, Clayberger C, Moody D, Do D, Remington JS, Talib S, Subauste CS (1996). Human CD4+ and CD8+ T lymphocytes are both cytotoxic to Toxoplasma gondii-infected cells. Infect Immun.

[16] Denkers EY, Gazzinelli RT (1998). Regulation and function of T-cell-mediated immunity during Toxoplasma gondii infection. Clin Microbiol Rev.

[17] Buscaglia CA, Coppens I, Hol WG, Nussenzweig V (2003). Sites of interaction between aldolase and thrombospondin-related anonymous protein in Plasmodium. Mol Biol Cell.

[18] Jewett TJ, Sibley LD (2003). Aldolase forms a bridge between cell surface adhesins and the actin cytoskeleton in apicomplexan parasites. Mol Cell.

[19] Kappe SH, Buscaglia CA, Bergman LW, Coppens I, Nussenzweig V (2004). Apicomplexan gliding motility and host cell invasion: overhauling the motor model. Trends Parasitol.

[20] Starnes GL, Coincon M, Sygusch J, Sibley LD (2009). Aldolase is essential for energy production and bridging adhesin-actin cytoskeletal interactions during parasite invasion of host cells. Cell Host Microbe.

[21] G-w Z, Shen B, Xie Q, Xu L-x, Yan R-f, Song X-k, Ibrahim Adam H, Li X-r (2012). Isolation and Molecular Characterization of Toxoplasma gondii from Chickens in China. Journal of Integrative Agriculture.

[22] Sasai K, Aita M, Lillehoj H, Miyamoto T, Fukata T, Baba E (2000). Dynamics of lymphocyte subpopulation changes in the cecal tonsils of chickens infected with Salmonella enteritidis. Vet Microbiol.

[23] Song H, Yan R, Xu L, Song X, Shah MA, Zhu H, Li X (2010). Efficacy of DNA vaccines carrying Eimeria acervulina lactate dehydrogenase antigen gene against coccidiosis. Exp Parasitol.

[24] Meng M, Zhou A, Lu G, Wang L, Zhao G, Han Y, Zhou H, Cong H, Zhao Q, Zhu X-Q (2013). DNA prime and peptide boost immunization protocol encoding the Toxoplasma gondii GRA4 induces strong protective immunity in BALB/c mice. BMC Infect Dis.

[25] Zhang N-Z, Huang S-Y, Zhou D-H, Chen J, Xu Y, Tian W-P, Lu J, Zhu X-Q (2013). Protective immunity against Toxoplasma gondii induced by DNA immunization with the gene encoding a novel vaccine candidate: calcium-dependent protein kinase 3. BMC Infect Dis.

[26] Zhao G, Zhou A, Lv G, Meng M, Sun M, Bai Y, Han Y, Wang L, Zhou H, Cong H (2013). Toxoplasma gondii cathepsin proteases are undeveloped prominent vaccine antigens against toxoplasmosis. BMC Infect Dis.

[27] Moreno S, Timon M (2004). DNA vaccination: an immunological perspective. Inmunologia.

[28] Chardes T, Bourguin I, Mevelec M, Dubremetz J, Bout D (1990). Antibody responses to Toxoplasma gondii in sera, intestinal secretions, and milk from orally infected mice and characterization of target antigens. Infect Immun.

[29] Pinon J, Toubas D, Marx C, Mougeot G, Bonnin A, Bonhomme A, Villaume M, Foudrinier F, Lepan H (1990). Detection of specific immunoglobulin E in patients with toxoplasmosis. J Clin Microbiol.

[30] Macpherson A, McCoy K, Johansen F, Brandtzaeg P (2008). The immune geography of IgA induction and function. Mucosal Immunol.

[31] Woof JM, Kerr MA (2006). The function of immunoglobulin A in immunity. J Pathol.

[32] Couper KN, Roberts CW, Brombacher F, Alexander J, Johnson LL (2005). Toxoplasma gondii-specific immunoglobulin M limits parasite dissemination by preventing host cell invasion. Infect Immun.

[33] Kaneko Y, Takashima Y, Xuaun X, Igarashi I, Nagasawa H, Mikami T, Otsuka H (2004). Natural IgM antibodies in sera from various animals but not the cat kill Toxoplasma gondii by activating the classical complement pathway. Parasitology.

[34] Konishi E, Nakao M (1992). Naturally occurring immunoglobulin M antibodies: enhancement of phagocytic and microbicidal activities of human neutrophils against Toxoplasma gondii. Parasitology.

[35] Correa D, Cañedo‒Solares I, Ortiz‒Alegría L, Caballero‒Ortega H, Rico‒Torres C (2007). Congenital and acquired toxoplasmosis: diversity and role of antibodies in different compartments of the host. Parasite Immunol.

[36] Wong S, Hajdu M, Ramirez R, Thulliez P, McLeod R, Remington J (1993). Role of specific immunoglobulin E in diagnosis of acute toxoplasma infection and toxoplasmosis. J Clin Microbiol.

[37] Godard I, Darcy F, Deslee D, Dessaint J, Capron A (1990). Isotypic profiles of antibody responses to Toxoplasma gondii infection in rats and mice: kinetic study and characterization of target antigens of immunoglobulin A antibodies. Infect Immun.

[38] Dupont CD CD, Hunter CA (2012). Immune response and immunopathology during toxoplasmosis. Seminars in immunopathology.

[39] Janssen R, van Wengen A, Verhard E, de Boer T, Zomerdijk T, Ottenhoff TH, van Dissel JT (2002). Divergent role for TNF-α in IFN-γ-induced killing of Toxoplasma gondii and Salmonella typhimurium contributes to selective susceptibility of patients with partial IFN-γ receptor 1 deficiency. J Immunol.

[40] Silva NM, Vieira JCM, Carneiro CM, Tafuri WL (2009). Toxoplasma gondii: The role of IFN-gamma, TNFRp55 and iNOS in inflammatory changes during infection. Exp Parasitol.

[41] Takács AC, Swierzy IJ, Lüder CG (2012). Interferon-γ restricts Toxoplasma gondii development in murine skeletal muscle cells via nitric oxide production and immunity-related GTPases. PLoS One.

[42] Yarovinsky F (2014). Innate immunity to Toxoplasma gondii infection. Nat Rev Immunol.

[43] Angus C, Klivington-Evans D, Dubey J, Kovacs JA (2000). Immunization with a DNA plasmid encoding the SAG1 (P30) protein of Toxoplasma gondii is immunogenic and protective in rodents. J Infect Dis.

[44] Fachado A, Rodriguez A, Angel SO, Pinto DC, Vila I, Acosta A, Amendoeira RR, Lannes-Vieira J (2003). Protective effect of a naked DNA vaccine cocktail against lethal toxoplasmosis in mice. Vaccine.

[45] Mévélec M-N, Bout D, Desolme B, Marchand H, Magné R, Bruneel O, Buzoni-Gatel D (2005). Evaluation of protective effect of DNA vaccination with genes encoding antigens GRA4 and SAG1 associated with GM-CSF plasmid, against acute, chronical and congenital toxoplasmosis in mice. Vaccine.

[46] Scorza T, D'souza S, Laloup M, Dewit J, De Braekeleer J, Verschueren H, Vercammen M, Huygen K, Jongert E (2003). A GRA1 DNA vaccine primes cytolytic CD8+ T cells to control acute Toxoplasma gondii infection. Infect Immun.

[47] Meng M, He S, Zhao G, Bai Y, Zhou H, Cong H, Lu G, Zhao Q, Zhu X-Q (2012). Evaluation of protective immune responses induced by DNA vaccines encoding Toxoplasma gondii surface antigen 1 (SAG1) and 14-3-3 protein in BALB/c mice. Parasit Vectors.

[48] Alexander J, Jebbari H, Bluethmann H, Brombacher F, Roberts C (1998). The role of IL-4 in adult acquired and congenital toxoplasmosis. Int J Parasitol.

[49] Roberts C, Ferguson D, Jebbari H, Satoskar A, Bluethmann H, Alexander J (1996). Different roles for interleukin-4 during the course of Toxoplasma gondii infection. Infect Immun.

[50] Khan AQ, Chen Q, Wu Z-Q, Paton JC, Snapper CM (2005). Both innate immunity and type 1 humoral immunity to Streptococcus pneumoniae are mediated by MyD88 but differ in their relative levels of dependence on toll-like receptor 2. Infect Immun.

[51] Lourenço EV, Bernardes ES, Silva NM, Mineo JR, Panunto-Castelo A, Roque-Barreira M-C (2006). Immunization with MIC1 and MIC4 induces protective immunity against Toxoplasma gondii. Microbes Infect.

[52] Passos ST, Silver JS, O'Hara AC, Sehy D, Stumhofer JS, Hunter CA (2010). IL-6 promotes NK cell production of IL-17 during toxoplasmosis. J Immunol.

[53] Chen Z, O'Shea JJ (2008). Th17 cells: a new fate for differentiating helper T cells. Immunol Res.

[54] Miyamoto M, Prause O, Sjöstrand M, Laan M, Lötvall J, Lindén A (2003). Endogenous IL-17 as a mediator of neutrophil recruitment caused by endotoxin exposure in mouse airways. J Immunol.

[55] Ye P, Rodriguez FH, Kanaly S, Stocking KL, Schurr J, Schwarzenberger P, Oliver P, Huang W, Zhang P, Zhang J (2001). Requirement of interleukin 17 receptor signaling for lung CXC chemokine and granulocyte colony-stimulating factor expression, neutrophil recruitment, and host defense. J Exp Med.

[56] Champsi J, Young L, Bermudez L (1995). Production of TNF-alpha, IL-6 and TGF-beta, and expression of receptors for TNF-alpha and IL-6, during murine Mycobacterium avium infection. Immunology.

[57] Hayashi H, Inoue Y, Tsutsui H, Okamura H, Nakanishi K, Onozaki K (2003). TGF down-regulates IFN- production in IL-18 treated NK cell line LNK5E6. Biochem Biophys Res Commun.

[58] Lin J, Seguin R, Keller K, Chadee K (1995). Transforming growth factor-beta 1 primes macrophages for enhanced expression of the nitric oxide synthase gene for nitric oxide-dependent cytotoxicity against Entamoeba histolytica. Immunology.

[59] Wan YY, Flavell RA (2008). TGF-β and regulatory T cell in immunity and autoimmunity. J Clin Immunol.

[60] Tenorio EP, Fernández J, Castellanos C, Olguín JE, Saavedra R (2011). CD4+ Foxp3+ regulatory T cells mediate Toxoplasma gondii‒induced T‒cell suppression through an IL‒2‒related mechanism but independently of IL‒10. Eur J Immunol.

[61] Dzierszinski F, Hunter C (2008). Advances in the use of genetically engineered parasites to study immunity to Toxoplasma gondii. Parasite Immunol.

[62] Henriquez FL, Woods S, Cong H, McLeod R, Roberts CW (2010). Immunogenetics of Toxoplasma gondii informs vaccine design. Trends Parasitol.

[63] Liesenfeld O, Kosek J, Remington JS, Suzuki Y (1996). Association of CD4+ T cell-dependent, interferon-gamma-mediated necrosis of the small intestine with genetic susceptibility of mice to peroral infection with Toxoplasma gondii. J Exp Med.

[64] Curiel T, Krug EC, Purner MB, Poignard P, Berens R (1993). Cloned human CD4+ cytotoxic T lymphocytes specific for Toxoplasma gondii lyse tachyzoite-infected target cells. J Immunol.

[65] Gazzinelli R, Xu Y, Hieny S, Cheever A, Sher A (1992). Simultaneous depletion of CD4+ and CD8+ T lymphocytes is required to reactivate chronic infection with Toxoplasma gondii. J Immunol.

[66] Hakim FT, Gazzinelli RT, Denkers E, Hieny S, Shearer G, Sher A (1991). CD8+ T cells from mice vaccinated against Toxoplasma gondii are cytotoxic for parasite-infected or antigen-pulsed host cells. J Immunol.

[67] Nakano Y, Hisaeda H, Sakai T, Zhang M, Maekawa Y, Zhang T, Nishitani M, Ishikawa H, Himeno K (2001). Granule‒dependent killing of Toxoplasma gondii by CD8+ T cells. Immunology.

[68] Khosroshahi KH, Ghaffarifar F, Sharifi Z, D’Souza S, Dalimi A, Hassan ZM, Khoshzaban F (2012). Comparing the effect of IL-12 genetic adjuvant and alum non-genetic adjuvant on the efficiency of the cocktail DNA vaccine containing plasmids encoding SAG-1 and ROP-2 of Toxoplasma gondii. Parasitol Res.

[69] Li J, Han Q, Gong P, Yang T, Ren B, Li S, Zhang X (2012). Toxoplasma gondii rhomboid protein 1 (TgROM1) is a potential vaccine candidate against toxoplasmosis. Vet Parasitol.

[70] Wang H, Liu Q, Liu K, Zhong W, Gao S, Jiang L, An N (2007). Immune response induced by recombinant Mycobacterium bovis BCG expressing ROP2 gene of Toxoplasma gondii. Parasitol Int.

[71] Casciotti L, Ely KH, Williams ME, Khan IA (2002). CD8 + −T-cell immunity against Toxoplasma gondii can be induced but not maintained in mice lacking conventional CD4+ T cells. Infect Immun.

[72] Purner MB, Berens RL, Nash PB, van Linden A, Ross E, Kruse C, Krug EC, Curiel TJ (1996). CD4-mediated and CD8-mediated cytotoxic and proliferative immune responses to Toxoplasma gondii in seropositive humans. Infect Immun.

[73] Pamer E, Cresswell P (1998). Mechanisms of MHC class I-restricted antigen processing. Annu Rev Immunol.

[74] Jerome K, Sloan D, Aubert M (2003). Measurement of CTL-induced cytotoxicity: the caspase 3 assay. Apoptosis.

[75] Keller P, Schaumburg F, Fischer SF, Häcker G, Groß U, Lüder CG (2006). Direct inhibition of cytochrome c‒induced caspase activation in vitro by Toxoplasma gondii reveals novel mechanisms of interference with host cell apoptosis. FEMS Microbiol Lett.

[76] Kim L, Denkers EY (2006). Toxoplasma gondii triggers Gi-dependent PI 3-kinase signaling required for inhibition of host cell apoptosis. J Cell Sci.

[77] Payne TM, Molestina RE, Sinai AP (2003). Inhibition of caspase activation and a requirement for NF-κB function in the Toxoplasma gondii-mediated blockade of host apoptosis. J Cell Sci.

[78] Marshall ES, Elshekiha HM, Hakimi M-A, Flynn RJ (2011). Toxoplasma gondii peroxiredoxin promotes altered macrophage function, caspase-1-dependent IL-1b secretion enhances parasite replication. Vet Res.

[79] Ewen C, Kane KP, Shostak I, Griebel PJ, Bertram EM, Watts TH, Bleackley R, McElhaney JE (2003). A novel cytotoxicity assay to evaluate antigen-specific CTL responses using a colorimetric substrate for Granzyme B. J Immunol Methods.

[80] He L, Hakimi J, Salha D, Miron I, Dunn P, Radvanyi L (2005). A sensitive flow cytometry-based cytotoxic T-lymphocyte assay through detection of cleaved caspase 3 in target cells. J Immunol Methods.

